# Supplementary material for: Long-term stability of cortical ensembles
Source: eLife. 2021 Jul 30;10:e64449. doi: 10.7554/eLife.64449 (PMC8376248; doi:10.7554/eLife.64449)
Supplement: Figure 3—source data 2. [file elife-64449-fig3-data2.docx]

**Figure 3 — Table 2. Mice and recording days from Allen Brain Observatory Visual Coding dataset.**

| **Mouse #** | **Sex** | **GCaMP** | **PD at day 1** | **Container ID** | **Experiment IDs** | **Day 1** | **Day 2-5** | | | | **Day 6-8** | | |
| --- | --- | --- | --- | --- | --- | --- | --- | --- | --- | --- | --- | --- | --- |
|  |  |  |  |  |  | **1** | **2** | **3** | **4** | **5** | **6** | **7** | **8** |
| **1** | **♂** | **6f** | **80** | 650389885 | 650389887 - 650512363 - 652094901 | x | x |  |  |  |  |  | x |
| **2** | **♂** | **6f** | **90** | 652842570 | 652842572 - 652988777- 653053207 | x |  |  | x | x |  |  |  |
| **3** | **♂** | **6f** | **99** | 653125128 | 653125130 - 653552163 - 653923501 | x |  |  |  |  | x | x |  |
| **4** | **♂** | **6f** | **95** | 661437138 | 661437140 - 662351346 - 662358233 | x | x | x |  |  |  |  |  |
| **5** | **♂** | **6f** | **96** | 661732156 | 661753184 - 662989044 - 663485329 | x |  |  |  |  | x | x |  |
| **6** | **♂** | **6f** | **111** | 666589599 | 666589601 - 667011230 - 667364442 | x | x |  |  | x |  |  |  |
| **7** | **♀** | **6f** | **85** | 680156909 | 680156911 - 681673022 - 682054669 | x |  |  |  |  | x | x |  |
| Mice: Slc17a7-IRES2-Cre::Camk2a-tTA::Ai93 (Vglut1); structure: VISp; depth: 175 µm.  Source: Allen Brain Observatory Visual Coding (<http://observatory.brain-map.org/visualcoding>) | | | | | | | | | | | | | |
