# Supplementary material for: Long-term stability of cortical ensembles
Source: eLife. 2021 Jul 30;10:e64449. doi: 10.7554/eLife.64449 (PMC8376248; doi:10.7554/eLife.64449)
Supplement: Figure 3—source data 3. [file elife-64449-fig3-data3.docx]

**Figure 3 — Table 3. Mice from Churchland Lab dataset.**

| **Mouse #** | **GCaMP** | **ID** | **Date ID** |
| --- | --- | --- | --- |
| **1** | **6f** | **mSM74** | 2 Oct 2018 |
| **2** | **6f** | **mSM75** | 3 Oct 2018 |
| **3** | **6f** | **RS02** | 15 Nov 2018 |
| **4** | **6f** | **RS04** | 13 Nov 2018 |
| Mice: Ai93::Emx-Cre::LSL-tTA::CaMK2α-tTA; structure: V1; depth: 150-450 μm.  Source: Churchland Lab (Musal et al., 2019) | | | |
